# Proteomic Profiling of *Bifidobacterium bifidum* S17 Cultivated Under *In Vitro* Conditions

**DOI:** 10.3389/fmicb.2016.00097

**Published:** 2016-02-12

**Authors:** Xiao Wei, Simiao Wang, Xiangna Zhao, Xuesong Wang, Huan Li, Weishi Lin, Jing Lu, Daria Zhurina, Boxing Li, Christian U. Riedel, Yansong Sun, Jing Yuan

**Affiliations:** ^1^Institute of Disease Control and Prevention, Academy of Military Medical SciencesBeijing, China; ^2^Institute of Microbiology and Biotechnology, University of UlmUlm, Germany

**Keywords:** proteomic profiling, metabolic pathways, pilin proteins, LC-MS/MS, *Bifidobacterium bifidum*

## Abstract

Bifidobacteria are frequently used in probiotic food and dairy products. *Bifidobacterium bifidum* S17 is a promising probiotic candidate strain that displays strong adhesion to intestinal epithelial cells and elicits potent anti-inflammatory capacity both *in vitro* and in murine models of colitis. The recently sequenced genome of *B. bifidum* S17 has a size of about 2.2 Mb and encodes 1,782 predicted protein-coding genes. In the present study, a comprehensive proteomic profiling was carried out to identify and characterize proteins expressed by *B. bifidum* S17. A total of 1148 proteins entries were identified by liquid chromatography coupled to tandem mass spectrometry (LC-MS/MS), representing 64.4% of the predicted proteome. 719 proteins could be assigned to functional categories according to cluster of orthologous groups of proteins (COGs). The COG distribution of the detected proteins highly correlates with that of the complete predicted proteome suggesting a good coverage and representation of the genomic content of *B. bifidum* S17 by the proteome. COGs that were highly present in the proteome of *B. bifidum* S17 were Translation, Amino Acid Transport and Metabolism, and Carbohydrate Transport and Metabolism. Complete sets of enzymes for both the bifidus shunt and the Embden-Meyerh of pathway were identified. Further bioinformatic analysis yielded 28 proteins with a predicted extracellular localization including 14 proteins with an LPxTG-motif for cell wall anchoring and two proteins (elongation factor Tu and enolase) with a potential moonlighting function in adhesion. Amongst the predicted extracellular proteins were five of six pilin proteins encoded in the *B. bifidum* S17 genome as well as several other proteins with a potential role in interaction with host structures. The presented results are the first compilation of a proteomic reference profile for a *B. bifidum* strain and will facilitate analysis of the molecular mechanisms of physiology, host-interactions and beneficial effects of a potential probiotic strain.

Bifidobacteria represent an important group of the human intestinal microbiota (Bottacini et al., 2014). Due to their reported ability to reduce cholesterol levels, exclude intestinal pathogens, strengthen the intestinal barrier, alleviate symptoms of constipation, and/or modulate the immune response they are frequently used as active ingredients in food and dairy-based products (Leahy et al., 2005; Gareau et al., 2010). In order to reveal the molecular mechanisms responsible for these beneficial effects, several bifidobacterial strains have recently been sequenced (Ventura et al., 2009). Analysis of bifidobacterial genomes has led to the identification of various structures involved in host colonization and probiotic properties. For example, *Bifidobacterium breve* UCC2003 was shown to encode genes for production of exopolysaccharides (Fanning et al., 2012). These exopolysaccharides support persistence in the murine gastrointestinal tract and are required for the protective effect of *B. breve* UCC2003 against infections with the murine pathogen *Citrobacter rodentium*. *B. breve* UCC2003 also possesses type IV tight adherence pili and these pili were shown to support long term colonization of mice (O’Connell Motherway et al., 2011). Similarly, other strains and species of bifidobacteria were shown to contain gene clusters for Tad and/or sortase-dependent pili for some of which interaction with host structures has been shown (Foroni et al., 2011; Turroni et al., 2013, 2014b).

*Bifidobacterium bifidum* strains belong to the infant-type bifidobacteria and show a remarkable adaptation to their ecological niche in the intestinal tract of human neonates. This includes a large number of adhesive structures and a specific ability to utilize host-derived glycans (Turroni et al., 2014a). *B. bifidum* S17 was isolated from feces of a breast-fed infant and displays unusually strong adhesion to IECs (Riedel et al., 2006a; Preising et al., 2010; Gleinser et al., 2012). Additionally, the strain elicits a promising anti-inflammatory capacity both *in vitro* (Riedel et al., 2006b; Preising et al., 2010) and *in vivo* in three different murine models of colitis (Preising et al., 2010; Philippe et al., 2011; Grimm et al., 2015). The genome of *B. bifidum* S17 was sequenced and annotated and contains a predicted 1,782 protein-coding ORFs (Zhurina et al., 2011) including one Tad and three sortase-dependent pili gene clusters as well as several other genes suspected or shown to play a role in adhesion to host structures or host colonization (Gleinser et al., 2012; Westermann et al., 2012).

In recent years, proteomic analysis has become an indispensible tool to analyze the biology of microorganisms, their response to changes in the environmental conditions and their interaction with the host (Otto et al., 2014). One of first the reports on a proteomic analysis of a *Bifidobacterium* sp. strain was a proteomic reference map obtained by 2D electrophoresis and MALDI TOF-TOF mass spectrometry (Yuan et al., 2006). Since then, this technique has been used to study adaptation of various bifidobacteria to bile and oxidative stress (Sánchez et al., 2007; Xiao et al., 2011) and different carbon sources (Liu et al., 2011, 2015), host-induced proteome changes of *B. longum* NCC2705 (Yuan et al., 2008) or its interaction with IECs (Wei et al., 2014). Turroni et al. (2010) performed a proteomic analysis of *B. bifidum* PRL2010 during growth on different sugars and following contact with cultured epithelial cells (Turroni et al., 2010).

However, proteomic approaches that include 2D electrophoresis have limitations in the detection of alkaline and low-abundance proteins (Otto et al., 2014). These limitations can be overcome by 1D gel-based LC-MS/MS (Wickramasekara et al., 2011; Otto et al., 2014). Moreover, LC-MS/MS is more efficient and accurate when analyzing differential global protein expression quantitatively and was used for proteomics of *Burkholderia vietnamiensis* (Wickramasekara et al., 2011). Another example is the analysis of secretion profiles of *B. pseudomallei* MSHR668 (Burtnick et al., 2014). LC-MS/MS has also been employed for comparative proteomics of two *Lactobacillus rhamnosus* strains (Savijoki et al., 2011). For bifidobacteria, LC-MS/MS was used for differential proteomics of two *B. longum* strains (Guillaume et al., 2009) and a proteomic profiling of *B. longum* subsp. *infantis* during growth on lactose, glucose, and galacto-, fructo-, and human milk oligosaccharides (Kim et al., 2013).

In the present study, the proteome of *B. bifidum* S17 grown *in vitro* was analyzed by 1D gel-based ultra-high performance LC-MS/MS representing the first comprehensive proteomic profile for the species *B. bifidum*.

## Materials and Methods

### Bacterial Strains and Growth Conditions

*Bifidobacterium bifidum* S17 was cultured in sealed jars anaerobically at 37°C in *Lactobacillus* MRS medium (Difco) supplemented with 0.05% L-cysteine. Anaerobic conditions were achieved and maintained using AnaeroGen sachets (Thermo Scientific). Bacteria were harvested for proteomic analysis in mid-exponential growth phase at an optical density at 600 nm of 0.9 corresponding to approximately 1.5 × 10^8^ colony forming units/ml.

### Preparation of Whole Cell Protein Extracts

Bacteria were harvested by centrifugation, washed twice in phosphate-buffered saline (PBS), and pellets (about 0.30 g) were resuspended in 5 mL of lysis buffer (8 M urea, 30 mM HEPES, 1 mM phenylmethylsulfonyl fluoride, 2 mM ethylenediaminetetraacetic acid, 10 mM dithiothreitol) containing one dissolved tablet of complete protease inhibitor (Roche Diagnostics, Mannheim, Germany). Bacteria were then sonicated for 10 min on ice using a Sonifier 750 (Branson Ultrasonics Corp., Danbury, CT, USA) with the following conditions: 2 s of sonication with a 3 s interval set at 25% duty cycle. The cell lysate suspension was centrifuged for 30 min at 20,000 × *g* to collect the supernatant. The proteins were reduced with 10 mM dithiothreitol at 56 °C for 1 h, and alkylated with 55 mM iodoacetamide at room temperature for 1 h in the dark. The treated proteins were precipitated in acetone at –20°C for 3 h. After centrifugation at 20,000 × *g* for 30 min, the protein pellet was resuspended and ultrasonicated in pre-chilled 50% tetraethylammonium bromide buffer with 0.1% sodium dodecyl sulfate (SDS). The proteins were regained after centrifugation at 20,000 × *g* and the protein concentrations were measured by Bradford assay.

### 1D SDS Polyacrylamid Gelelectrophersis (SDS-PAGE) and in-Gel Digestion

The proteome was analyzed by 1D gel-based LC-MS/MS as described previously (Albrethsen et al., 2010). In brief, the proteins were separated via SDS polyacrylamid gelelectrophersis (SDS-PAGE) using precast 4–12 % gradient gels containing Bis-Tris buffer (NuPAGE MES system, Invitrogen). Protein gels were run by using 1x running buffer [259 mM Tris base, 2 M glycine, 1% (w/v) SDS in ddH_2_O] at a constant voltage of 85 V for 20 min followed by 150 V for 40 min (until the dye runs off the gel). The gels were then stained with Coomassie R-250. A single lane of stained gel was cut into 10 pieces of approximately the same size and transferred into 1.5 mL Eppendorf tubes to perform in-gel tryptic digestion. Gel bands were destained and tryptically digested as described previously (Yuan et al., 2006). The digested peptides were extracted with 50% acetonitrile and 2% formic acid solution. After extraction, the peptides were transferred into 500 μL Eppendorf tubes and concentrated using a Speed-Vac Concentrator (Savant) and a volume of 10 μL containing 15 μg of protein was loaded into individual high-performance liquid chromatography (HPLC) autosampler vials and analyzed by liquid chromatography-tandem mass spectrometry (LC-MS/MS).

### LC-MS/MS Analysis

Peptide samples were analyzed by ultra-high performance LC-MS/MS on a quadrupole-Orbitrap mass spectrometer (Q-Exactive; Thermo Fisher, Germany) equipped with 15 cm (length) by 75 μm (inside diameter) column packed with 5 μm C18 medium (Thermo Fisher) which was kept at 21°C throughout the analysis. Mobile phase A was MilliQ water with 0.1% (v/v) formic acid. Mobile phase B was 99.9% (v/v) acetonitrile, 0.1% acetic acid. Gradient was run from 0% B to 30% B over 40 min and then to 80% B for 15 min. The LC was interfaced to a quadrupole-Orbitrap mass spectrometer (Q-Exactive; Thermo Fisher) via nano-electrospray ionization. An electrospray voltage of 1.8 kV was applied. The mass spectrometer was programmed to acquire, by data-dependent acquisition, tandem mass spectra from the top 20 ions in the full scan from 350 to 2,000 m/z. Dynamic exclusion was set to 15 s, singly charged ions were excluded, the isolation width was set to 2 m/z, the full MS resolution was set to 70,000, and the MS/MS resolution was set to 17,500. Normalized collision energy was set to 28, automatic gain control to 1e6, maximum fill MS to 20 ms, maximum fill MS/MS to 60 ms, and the under fill ratio to 0.1%.

### Protein Identification and Bioinformatic Analysis

Peptide identification were performed using Mascot v2.3.01 (Matrix Science Ltd.)^1^ licensed in-house^2^ (Albrethsen et al., 2010). Peptide mass finger printing searches were performed using Mascot v2.2.06 (Matrix Science Ltd.) licensed in-house. Monoisotopic peptide masses were used to search the databases, allowing a peptide mass accuracy of 30 ppm and fragment ion tolerance of 0.2 Dalton. Both methionine oxidation and cysteine carboxyamidomethylation were considered in the process. For protein identification by peptide mass fingerprinting, peptide masses were searched against the publically available database for *B. bifidum* S17 (NCBI Reference Sequence: NC_014616.1). For unambiguous identification of proteins, more than five peptides must be matched and the sequence coverage must be greater than 15%. The complete proteomic dataset is deposited and publically accessible on the iProX database^3^ under project number IPX00067300.

Calculation of protein molecular weights and isoelectric points were carried out by the protparam software from the Expasy toolbox^4^. Functional classification of identified proteins was performed by BLASTPGP (Altschul et al., 1997) searching against the databases of Cluster of Orthologous Groups (COGs^5^) (Tatusov et al., 2000). The cellular localizations of all identified proteins were predicted by PSORTb version 3.0 (Yu et al., 2010). Prediction of signal peptides was carried out with SignalP Version 4.1 (Petersen et al., 2011), TMHMM server 2.0 (Sonnhammer et al., 1998) was used to predict transmembrane helices, and LocateP database (Zhou et al., 2008) was used for prediction of cell wall and lipid anchor motifs.

## Results

The 2.2-Mb genome of *B. bifidum* S17 contains 1,782 predicted protein-coding ORFs. To obtain an overview on the proteins expressed by *B. bifidum* S17 under standard laboratory conditions, whole protein extracts of bacteria grown in MRS medium were subjected to LC-MS/MS and protein identification. A total of 1,148 proteins were successfully matched unambiguously to one of the 1,782 predicted proteins of *B. bifidum* S17, i.e., a coverage of 64.4% of the complete predicted proteome. All detected proteins the peptide data matches the protein sequence as predicted in the genome annotation (data not shown). Moreover, the proteome contained a total of 235 (conserved) hypothetical proteins demonstrating that these proteins are actually expressed by *B. bifidum* S17 at least under *in vitro* conditions, which changes their status from “hypothetical” to proteins with unknown function.

**FIGURE 1:**
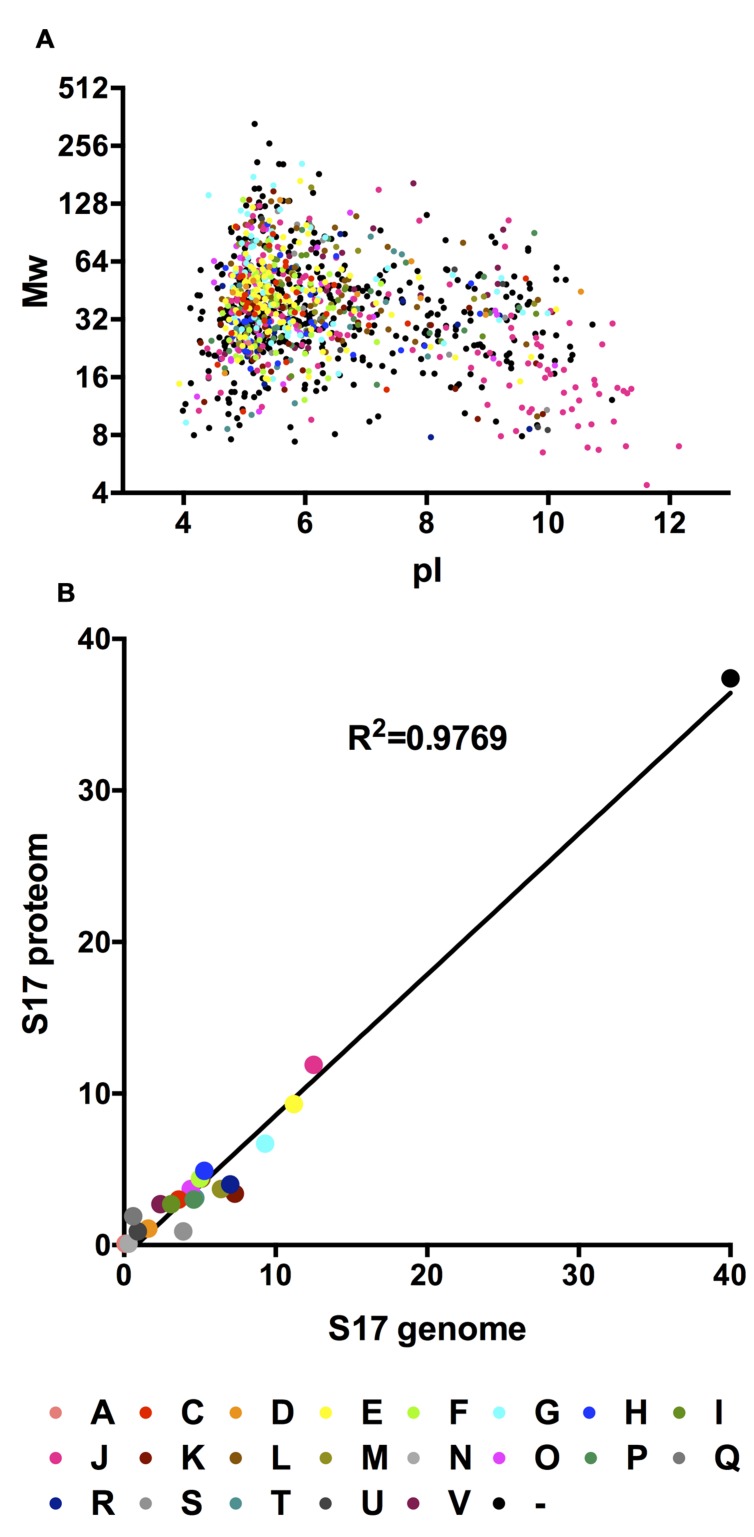
**(A)** *In silico* 2D gel electrophoresis of the identified proteins of the *B. bifidum* S17 proteome. For each identified protein, calculated molecular weight was plotted against the calculated pI. **(B)** Correlation analysis of the COG distributions of the detected proteins in the proteome and all proteins encoded on the genome of *B. bifidum* S17.

**Supplementary Table S1** summarizes these proteins along with information on predicted pI, molecular mass, function, signal peptides, and subcellular location. 76.7% of the *B. bifidum* S17 proteins detected by LC-MS/MS are acidic with pI between 3 and 7, 12.5% have a pI between 7 and 9, whereas 10.8% of the proteins have a pI greater than 9 (**Figure 1A**). Classification of the 1,148 detected proteins into clusters of orthologous groups of proteins (COGs) assigned 719 proteins into 22 functional categories (**Table 1**). The remaining 429 unclassified proteins (37,4%) were denoted as COG-absent proteins. For none of the COGs an obvious clustering according to pI and Mw could be observed (**Figure 1A**). Comparison of the proteomes and genomes of *B. bifidum* S17 that the COG distribution of the *B. bifidum* S17 proteome is highly correlated to the distribution of all 1,782 predicted protein-coding ORFs of the genome (**Figure 1B**; coefficient of determination *R*^2^ = 0.9769). Also, none of the COGs is skewed markedly off the regression in the proteome/genome comparison. Thus, none of the COGs seems to be over- or underrepresented in the proteome compared to the genome. Collectively, this indicates that the proteome captured by LC-MS/MS is comprehensive and covers all functional categories to a similar extend as encoded by the genomic information of *B. bifidum* S17.

**Table 1:** Relative abundance of functional categories according to clusters of orthologous groups of proteins (COGs) in the *Bifidobacterium bifidum* S17 proteome.

COG functional category	Abundance
A: RNA processing and modification	0.1%
C: Energy production and conversion	3.0%
D: Cell cycle control, mitosis and meiosis	1.1%
E: Amino acid transport and metabolism	9.3%
F: Nucleotide transport and metabolism	4.4%
G: Carbohydrate transport and metabolism	6.7%
H: Coenzyme transport and metabolism	4.9%
I: Lipid transport and metabolism	2.7%
J: Translation	11.9%
K: Transcription	3.4%
L: Replication, recombination and repair	4.4%
M: Cell wall/membrane biogenesis	3.7%
N: Cell motility	0.1%
O: Posttranslational modification, protein turnover, chaperones	3.7%
P: Inorganic ion transport and metabolism	3.0%
Q: Secondary metabolites biosynthesis, transport and catabolism	1.9%
R: General function prediction only	4.0%
S: Function unknown	0.9%
T: Signal transduction mechanisms	3.1%
U: Intracellular trafficking and secretion	0.9%
V: Defense mechanisms	2.7%
X: Transposons	0.2%
–: Not in COGs	37.4%

The functional categories with highest representation amongst the detected proteins were Translation (functional category J; 137 proteins, 11.9% of all detected proteins), Amino acid Transport and Metabolism (functional category E; 107 proteins, 9.3%), and Carbohydrate Transport and Metabolism (functional category G; 77 proteins, 6.7%). Collectively, 26% of all detected proteins of the *B. bifidum* S17 proteome are assigned to functional categories J, A, K, and L. Thus, proteins possessing biological functions related to information storage, DNA replication, recombination and repair, RNA processing, transcription, and translation were highly prevalent in the proteome of *B. bifidum* S17. Of note, elongation factors Tu (EF-Tu; BBIF_1251) was amongst these proteins.

**FIGURE 2:**
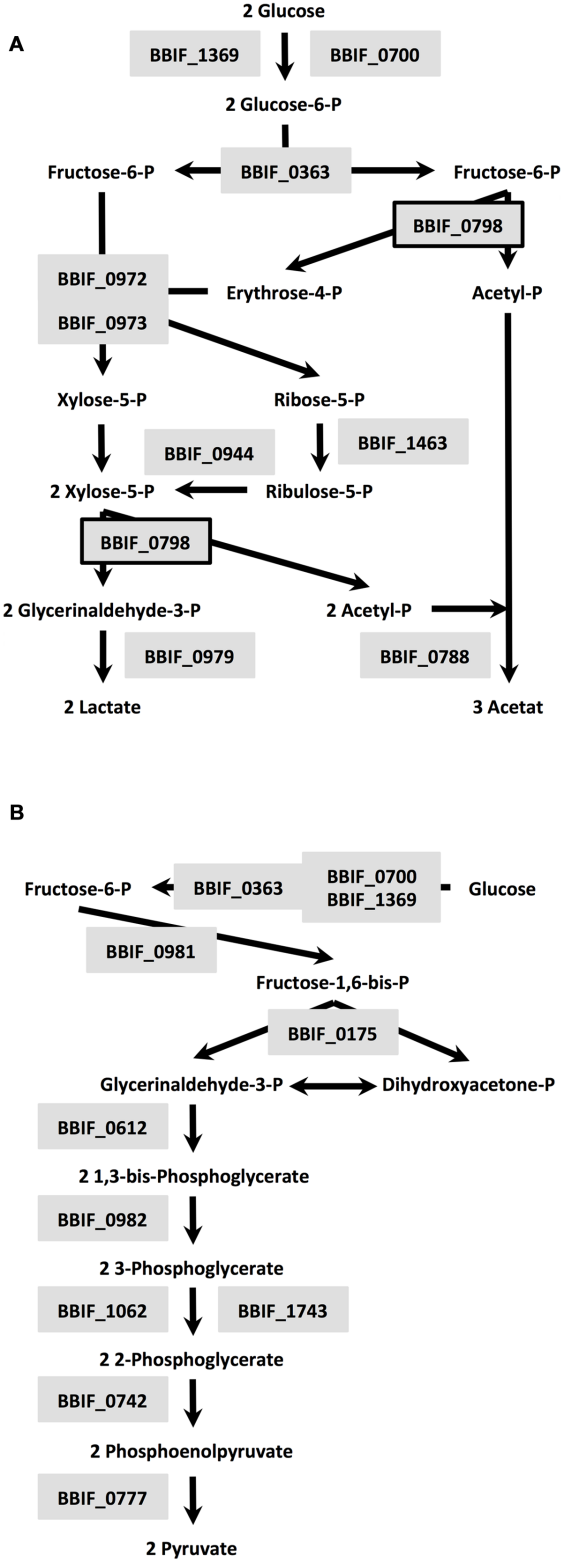
**Enzymes of the bifidus shunt **(A)** and Embden-Meyerhof pathway **(B)** detected in the proteome of *B. bifidum* S17: BBIF_1369: glucokinase; BBIF_0700: alternative glucokinase; BBIF_0798: xylulose-5-phosphate/fructose-6-phosphate phosphoketolase; BBIF_0363: glucose-6-phosphate isomerase; BBIF_0973: transaldolase; BBIF_0972: transketolase, BBIF_1463: probable ribose-5-phosphate isomerase; BBIF_0944: ribulosephosphate 3-epimerase; BBIF_0788: acetate kinase; BBIF_0979: L-lactate dehydrogenase; BBIF_0175: fructose-bisphosphate aldolase; BBIF_0981: triosephosphate isomerase; BBIF_0612: glyceraldehyde 3-phosphate dehydrogenase; BBIF_0982: phosphoglycerate kinase; BBIF_1062: phosphoglycerate mutase; BBIF_1743: phosphoglycerate mutase family protein; BBIF_0742: enolase; BBIF_0777: pyruvate kinase**.

A total of 9.3% of the proteome have a (predicted) role in amino acid transport, biosynthesis, urea cycle, and metabolism of amino groups including 14 aminotransferases (**Table 2**) and 6.7% of all identified proteins are related to carbohydrate transport and metabolism. The major and characteristic metabolic pathway of bifidobacteria for fermentation of hexose sugars is the so-called fructose-6-phosphate or “bifid” shunt (Pokusaeva et al., 2011). We successfully detected a complete set of ten enzymes of the bifidus shunt (**Figure 2A**) including the key enzyme of the pathway xylulose-5-phosphate/fructose-6-phosphate phosphoketolase (BBIF_0798). Moreover, 11 enzymes for a complete Embden-Meyerhof pathway were identified (**Figure 2B**).

**Table 2:** Aminotransferases identified in the proteome of *B. bifidum* S17.

Locus_tag	Gene name	Description	Length [aa]^a^	MW [kDa]^b^
BBIF_0278	*aspC1*	Aminotransferase	401	42.8
BBIF_0311	*yhdR*	Aspartate aminotransferase	396	43.2
BBIF_0342	*bbif_0342*	Multiple substrate aminotransferase (MsaT) containing domain of GntR family (transcriptional regulator)	509	55.6
BBIF_0469	*bbif_0469*	Aspartate/tyrosine/aromatic aminotransferase	398	43.7
BBIF_0550	*hisC*	Histidinol-phosphate aminotransferase	391	43.2
BBIF_0701	*bbif_0701*	Aminotransferase	370	40
BBIF_0741	*ilvE1*	Branched-chain amino acid aminotransferase	262	29.1
BBIF_0863	*ilvE2*	Branched-chain amino acid aminotransferase	375	41.5
BBIF_0870	*bbif_0870*	Aminotransferase	522	58.1
BBIF_1100	*argD*	Acetylornithine aminotransferase	429	45.4
BBIF_1175	*bbif_1175*	Aspartate aminotransferase	394	43.5
BBIF_1428	*serC*	Phosphoserine aminotransferase	380	40.6
BBIF_1519	*bbif_1519*	N-succinyldiaminopimelate aminotransferase	392	41.5
BBIF_1610	*aspC2*	Aspartate aminotransferase	409	44.5

^a^*aa, amino acids.*^b^*kDa, kilo Dalton.*

**FIGURE 3:**
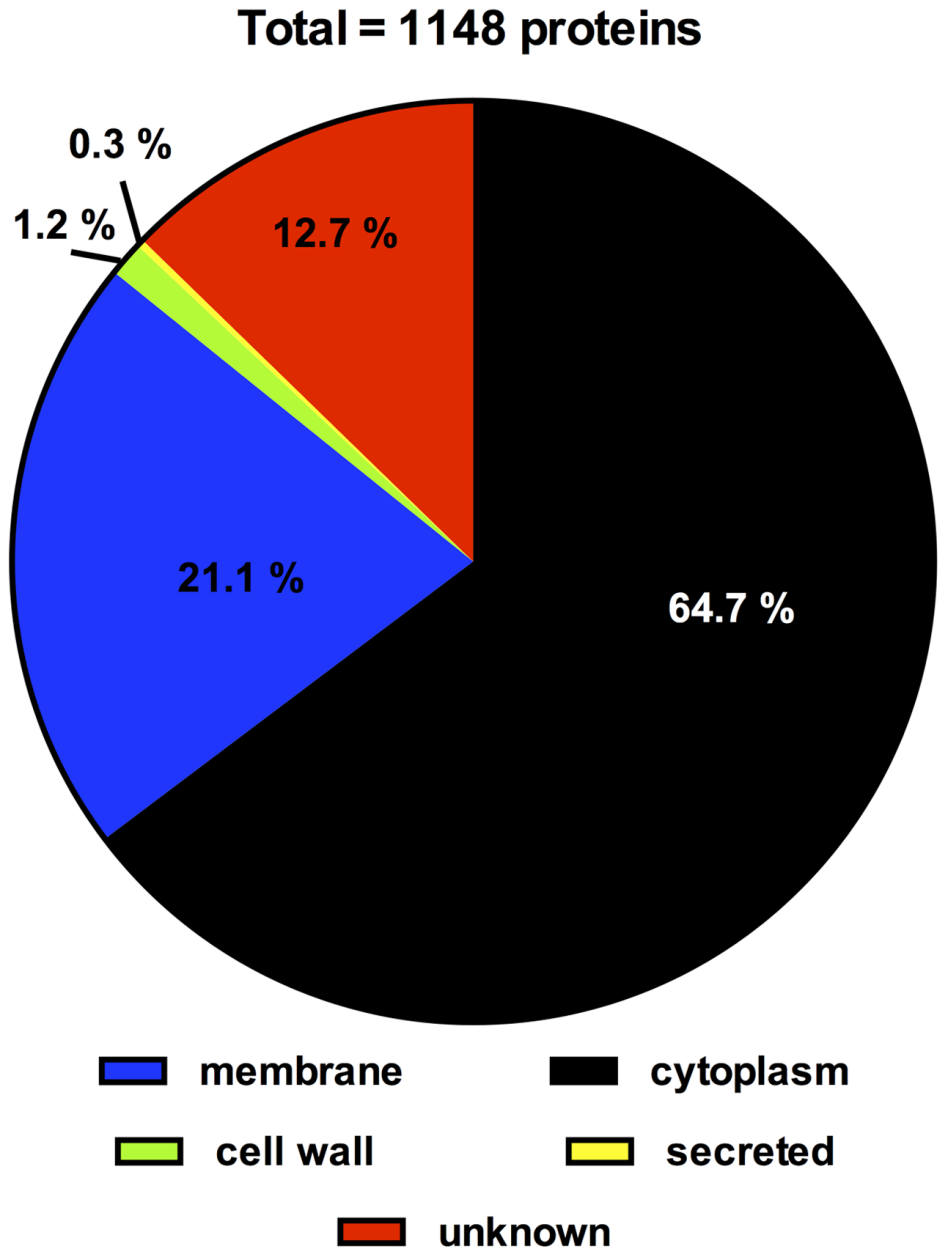
**Distribution of proteins of the *B. bifidum* S17 proteome according to subcellular location as predicted by PSORTb version 3.0 following correction for wrongly assigned localization of cell wall and extracellular proteins according to Table 3**.

Subcellular localization of all 1,148 proteins detected by LC-MS/MS was predicted by PSORTb Version 3.0. Extracellular and cell wall proteins play critical roles in establishing and maintaining interactions between a microbe and its environment. Thus, proteins which, according to the PSORTB prediction, are located in the cell wall or extracellular were further analyzed for transmembrane helices and signal peptides by SignalP v4.1 and TMHMM Server v2.0 and searched or LPxTG cell wall anchor motifs using the LocateP database to confirm their cellular localization (**Table 3**). Interestingly, these analysis suggest a different localization for several of these proteins as predicted by PSORTB. For example, three of the 28 proteins (BBIF_0312, BBIF_0337, BBIF_1026) contained none of these domains/sequence motifs and are thus probably cytoplasmatic. Following, corrections the PSORTB correction of the proteins in **Table 3**, the proteome of *B. bifidum* S17 consists of 743 (64.7%) cytoplasmic, 231 (21.1%) membrane, 14 (1.2%) cell wall, 3 (0.3%) extracellular proteins and 146 (12.7%) proteins with unknown cellular localization (**Figure 3**). A number of proteins of the *B. bifidum* S17 proteome with extracellular or cell wall localization have been associated with bacterial adhesion to host structures, colonization and/or immunomodulation. For example, five of the six pilin proteins for sortase-dependent pili including major pilins BBIF_0301 and BBIF_1761 and the minor pilins BBIF_0302; BBIF_1648, and BBIF_1761 were detected in the proteome. Further proteins identified in the proteome of *B. bifidum* S17 that may have a role in host colonization are the potential adhesin BBIF_0636 (BopA), the s subtilisin family peptidase BBIF_1681, and BBIF_1317 and BBIF_1734, two glycoside hydrolases of the fucosidase and sialidase family.

**Table 3:** Proteins of the *B. bifidum* S17 proteome with predicted cell wall or extracellular localization.

Locus_tag	Description	SP^a^	TMH^b^	Lipid anchor^c^	CW anchor^c^	Final prediction^d^
BBIF_0022	Alpha-L-arabinofuranosidase	1–36	13–35, 1138–1160	–	1133–1138 (LSHTG)	CW
BBIF_0285	Conserved hypothetical protein containing multiple sugar recognition domains	1–27	5–27, 1904–1926	–	1899–1903 (ISKTG)	CW
BBIF_0301	Conserved hypothetical protein containing von Willebrand factor type A domain	1–26	5–27, 1128–1150	–	1118–1123 (LPMTG)	CW
BBIF_0302	Conserved hypothetical protein with Cna B-type domain	1–29	7–29, 501–523	–	496–501 (LPKTG)	CW
BBIF_0507	Beta-galactosidase BbgIII	1–32	–	–	1903–1907 (LSKTG)	CW
BBIF_1317	Alpha-L-fucosidase	1–37	13–35, 1469–1491	–	1466–1470 (IAKTG)	CW
BBIF_1382	Conserved hypothetical protein containing bacterial Ig-like domain (group 2)	1–34	13–35, 1087–1106	–	1079–1083 (LSATG)	CW
BBIF_1461	Beta-N-acetylglucosaminidase	1–29	1935–1954	–	1926–1930 (ISKTG)	CW
BBIF_1576	Beta-N-acetylglucosaminidase	1–34	12–34, 1117–1139	–	1112–1116 (LSNTG)	CW
BBIF_1648	Conserved hypothetical protein containing CnaB domain and LPXTG-anchor	1–29	523–545	–	518–523 (LPLTG)	CW
BBIF_1681	Subtilisin family peptidase (lactocepin)	1–28	9–31,1325–1347	–	1320–1324 (VAKTG)	CW
BBIF_1734	Sialidase	1–35	13–35, 808–830	–	803–807 (LSKTG)	CW
BBIF_1761	Cell surface protein with gram positive anchor and Cna protein B-type domains	1–31	7–29, 505–527	–	500–505 (LPGTG)	CW
BBIF_1762	Cell surface protein with LPXTG anchor	–	2520–2542		2514–2519 (LPDTG)	CW
BBIF_0048	1,4-beta-N-acetylmuramidase	1–30	–	–	–	E
BBIF_0483	Conserved protein with the pectin lyase fold domain	1–30	–	–	–	E
BBIF_0522	Conserved hypothetical protein with CHAP domain	1–36	9–31	–	–	E
BBIF_0158	Trypsin-like serine protease	–	203–225	–	–	M
BBIF_0246	Peptidylprolylisomerase, FKBP-type	1–36	13–32	24–30 (VTLAACG)	–	M
BBIF_0313	Hypothetical protein BBIF_0313	–	236–258	–	–	M
BBIF_0592	Peptide/nickel transport system, substrate-binding protein	1–28	7–26	18–24 (ASLTACG)	–	M
BBIF_0636	Peptide/nickel transport system, extracellular solute-binding protein (BopA)	1–34	–	21–27 (LALGACG)	–	M
BBIF_1309	Peptide/nickel transport system, substrate-binding protein	–	55–77	–	–	M
BBIF_1426	Conserved hypothetical protein with NlpC/P60 domain	1–25	16–38	–	–	M
BBIF_1605	ABC transporter solute-binding protein	–	13–35	–	–	M
BBIF_0312	Conserved hypothetical protein	–	–	–	–	CP
BBIF_0337	Hsp20-family heat shock chaperone	–	–	–	–	CP
BBIF_1026	DNA polymerase III, delta subunit	–	–	–	–	CP

^a^*SP, signal peptide predicted using SignalP v4.1.*

^b^*TMH, transmembrane helices, predicted usingTMHMM Server v2.0.*

^c^*Lipid anchor and cell wall (CW) anchor motifs obtained from the LocateP database (http://www.cmbi.ru.nl/locatep-db/cgi-bin/locatepdb.py).*

^d^*final prediction for cellular localization cell wall (Cw), extracellular (E), membrane (M), orcytoplasma (Cp).*

## Discussion

*Bifidobacterium bifidum* strains belong to the infant-type bifidobacteria and some of these strains possess interesting properties related to host-health (Turroni et al., 2014a). In an attempt to compile the first proteomic reference profile for the species, *B. bifidum*, we determined the complete proteome of *B. bifidum* S17 grown in MRS, i.e., the standard medium for routine culture of bifidobacteria, by LC-MS/MS. In total 1,148 proteins were detected, i.e., a coverage 64.4% of all predicted proteins of *B. bifidum* S17. This is comparable to the coverage obtained by proteome analysis of two *L. rhamnosus* strains (Savijoki et al., 2011). However, it may still be an underestimation of the complete proteome of *B. bifidum* S17 expressed under the conditions tested. The experimental approach utilized the bacterial pellet of cultures grown in MRS. Thus, most proteins that are secreted into the cell culture supernatant are probably not captured. In line with the proteomes of other bacteria (Yuan et al., 2006; Liu et al., 2012), proteins with a function in DNA replication, recombination and repair, RNA processing, transcription, and translation to were highly prevalent in the proteome of *B. bifidum* S17. One of these proteins is elongation factors Tu (EF-Tu; BBIF_1251), which was previously shown to posses moonlighting function as an adhesin that mediates binding of *B. longum* NCC2705 and *Lactobacillus* sp. mucus, IECs, and/or ECM components of the host (Granato et al., 2004; Ramiah et al., 2008; Dhanani and Bagchi, 2013; Wei et al., 2014).

Detection of large numbers of enzymes of the bifidus shunt, glycolysis, and amino acid metabolism in the proteome of *B. bifidum* S17 confirms previous findings on the proteome of *B. longum* NCC2705 (Yuan et al., 2006). *B. bifidum* S17 was grown in MRS and *B. longum* NCC2705 in modified Garches medium for proteome analysis. Both media contain high levels of complex components such as yeast extract, peptone and/or beef extract and glucose as additional carbon source (De Man et al., 1960; Krzewinski et al., 1997). This may explain the high numbers of enzymes detected in the two proteomes involved in the uptake, degradation and fermentation of these substrates. Similar to EF-Tu, one of the proteins of the Embden-Meyerhof pathway is a cytoplasmic enzyme that has a moonlighting function. Enolase (BBIF_0742) was shown to mediate adhesion of *B. bifidum* and other bifidobacteria to IECs and components of the ECM (Candela et al., 2009; Wei et al., 2014). An interesting finding is the detection of 14 aminotransferases in the proteome (**Table 2**). Transamination reactions of amino acid converting pathways have recently attracted attention because they are the first step for the synthesis of important aroma compounds (Christensen et al., 1999) which may affect flavor of probiotic preparations.

A number of proteins of the *B. bifidum* S17 proteome contain domains and signal sequences for extracellular or cell wall location. Some of these proteins have been associated with bacterial adhesion to host structures, colonization and/or immunomodulation. Five of the six pilin proteins for sortase-dependent pili were identified in the proteome including major pilins BBIF_0301 and BBIF_1761 and the minor pilins BBIF_0302; BBIF_1648, and BBIF_1761. This suggests that at least two of the three sortase-dependent pili encoded on the genome of *B. bifidum* S17 are expressed under laboratory conditions. This is in line with the observation that sortase-dependent pili gene clusters are expressed by *B. bifidum* PRL2010 both *in vitro* and in the mouse GIT (Turroni et al., 2013) and pilus-like structures are detectable by atomic force microscopy in the same strain *in vitro* (Foroni et al., 2011). Expression of pili may have an important function in interaction of *B. bifidum* strains with the host. Heterologous expression of one of these gene clusters in *L. lactis* led to increased adhesion to ECM proteins and cultured IECs (Turroni et al., 2013). Moreover, the *L. lactis* strain expressing these pili elicits altered cytokine profiles in the murine gastrointestinal mucosa compared to the control. Another protein with potential role in adhesion to host structures is BBIF_0636. This protein is an extracellular solute-binding protein of a peptide/nickel transport system, which was also termed bifidobacterial outer protein A (BopA). Previous data obtained by us and others suggests that BopA mediates binding to cultured human IECs (Guglielmetti et al., 2008; Gleinser et al., 2012). However, these findings have been challenged recently (Kainulainen et al., 2013).

Three further proteins of the *B. bifidum* S17 proteome with a potential role in interaction with the host are BBIF_1317, BBIF_1681, and BBIF_1734. BBIF_1681 is a peptidase of the subtilisin family. The subtilisin family peptidase lactocepin of *L. casei* was shown to degrade pro-inflammatory cytokines contributing to the immunomodulatory effect of this probiotic bacterium (von Schillde et al., 2012). BBIF_1317 and BBIF_1734 are a glycoside hydrolases of the fucosidase and sialidase family, respectively. Sialidases are known as important virulence factors of bacterial pathogens that mediate attachment and degradation of host-derived mucus and were also shown to be involved in host colonization by commensal bacteria (Lewis and Lewis, 2012). Genes for mucin degradation pathways including sialidases are conserved amongst *B. bifidum* strains and most *B. bifidum* strains are able to grow on mucin as sole carbon source (Turroni et al., 2010). Similarly, fucosidases are involved in degradation of host-derived glucans such as human milk oligosaccharides and mucus (Turroni et al., 2010). Utilization of host-derived glucans is considered as nutritional adaptation to the intestinal tract of the (human) host (Turroni et al., 2010; Bottacini et al., 2014; Grimm et al., 2014). Thus, detection of proteins for degradation of host derived glycans under *in vitro* is somewhat surprising since these substrates are no present in standard growth medium, i.e., MRS. However, LC-MS/MS is a highly sensitive method that allows detection of even small amounts of a given protein (Otto et al., 2014). Moreover, pilin proteins that are only required *in vivo* were also present in the proteome of *B. bifidum* S17 and expression of pili genes and proteins *in vitro* has been observed previously (Foroni et al., 2011; Westermann et al., 2012). This suggests that regulation of host-interacting proteins may not strictly regulated in bifidobacteria.

In summary a total of 1,148 proteins of the predicted proteome were detected including important metabolic pathways, proteins known or suspected to be involved in adhesion and colonization as well as a large number of (previously) hypothetical proteins. This represents the first complete proteome analysis of the species *B. bifidum* and confirms previous findings on the proteome level.

## Author Contributions

JY, CR, and XW designed research; XW, DZ, HL, and WL performed research; XW, SW, BL, and XZ contributed new reagents or analytic tools; XW, JL, and CR analyzed data; XW, CR, and YS wrote the paper.

## Conflict of Interest Statement

The authors declare that the research was conducted in the absence of any commercial or financial relationships that could be construed as a potential conflict of interest.

## Footnotes

1 http://www.matrixscience.com/

2 http://www.proteomics.cn/

3 www.iprox.org

4 www.expasy.ch/tools/protparam.html

5 ftp://ftp.ncbi.nih.gov/pub/COG/COG2014/static/lists/listCOGs.html

## Supplementary Material

TABLE S1 **Characteristics of the 1,148 identified proteins**.

## Abbreviations

DTT: DL-dithiothreitol
ECM: extracellular matrix
EDTA: ethylene diamine tetraacetie acid
HEPES: 4-(2-hydroxyethyl)-1-piperazineethanesulfonic acid
IECs: intestinal epithelial cells
KEGG: Kyoto Encyclopedia of Genes and Genomes
LC-MS/MS: liquid chromatography coupled to tandem mass spectrometry
LPXTG: Leu-Pro-X-Thr-Gly
MRS: Man-Rogosa-Sharpe
ORF(s): open reading frame(s)
PMSF: phenylmethanesulfonyl fluoride
ThDP: thiamine diphosphate
TMDs: transmembrane domains
UHPLC: ultra-high-performance liquid chromatography
